# SARS-CoV-2 Nsp14 protein associates with IMPDH2 and activates NF-κB signaling

**DOI:** 10.3389/fimmu.2022.1007089

**Published:** 2022-09-13

**Authors:** Tai-Wei Li, Adam D. Kenney, Jun-Gyu Park, Guillaume N. Fiches, Helu Liu, Dawei Zhou, Ayan Biswas, Weiqiang Zhao, Jianwen Que, Netty Santoso, Luis Martinez-Sobrido, Jacob S. Yount, Jian Zhu

**Affiliations:** ^1^ Department of Pathology, The Ohio State University Wexner Medical Center, Columbus, OH, United States; ^2^ Department of Microbial Infection and Immunity, The Ohio State University Wexner Medical Center, Columbus, OH, United States; ^3^ Texas Biomedical Research Institute, San Antonio, TX, United States; ^4^ Department of Medicine, Columbia University Medical Center, New York, NY, United States; ^5^ Department of Genetics, The University of Alabama at Birmingham, Birmingham, AL, United States

**Keywords:** SARS-CoV-2, NF-κB, Nsp14, IL-8, IMPDH2, ribavirin, mycophenolic acid

## Abstract

Severe acute respiratory syndrome coronavirus 2 (SARS-CoV-2) infection leads to NF-κB activation and induction of pro-inflammatory cytokines, though the underlying mechanism for this activation is not fully understood. Our results reveal that the SARS-CoV-2 Nsp14 protein contributes to the viral activation of NF-κB signaling. Nsp14 caused the nuclear translocation of NF-κB p65. Nsp14 induced the upregulation of IL-6 and IL-8, which also occurred in SARS-CoV-2 infected cells. IL-8 upregulation was further confirmed in lung tissue samples from COVID-19 patients. A previous proteomic screen identified the putative interaction of Nsp14 with host Inosine-5’-monophosphate dehydrogenase 2 (IMPDH2), which is known to regulate NF-κB signaling. We confirmed the Nsp14-IMPDH2 protein interaction and identified that IMPDH2 knockdown or chemical inhibition using ribavirin (RIB) and mycophenolic acid (MPA) abolishes Nsp14- mediated NF-κB activation and cytokine induction. Furthermore, IMPDH2 inhibitors (RIB, MPA) or NF-κB inhibitors (bortezomib, BAY 11-7082) restricted SARS-CoV-2 infection, indicating that IMPDH2-mediated activation of NF-κB signaling is beneficial to viral replication. Overall, our results identify a novel role of SARS-CoV-2 Nsp14 in inducing NF-κB activation through IMPDH2 to promote viral infection.

## Introduction

SARS-CoV-2 is a beta-coronavirus that causes the current, severe COVID-19 pandemic globally. The viral genome of SARS-CoV-2 is a ~30 kb polycistronic, positive-strand RNA that encodes multiple structural and nonstructural proteins (1, 2). SARS-CoV-2 nonstructural proteins (Nsp 1-16) play diversified roles in supporting viral RNA/protein synthesis and virion assembly, including manipulating host gene expression and host antiviral responses (3, 4). It has been recently reported that SARS-CoV-2 infection suppresses type I interferon (IFN) signaling (5, 6), while it induces the activation of NF-κB signaling that plays a central role in the production of pro-inflammatory cytokines, including interleukin (IL)- 6 and IL-8 (5, 7, 8). In certain cases, massive inflammatory responses occur due to hyper-activation of the immune system, resulting in a widespread and uncontrolled cytokine storm, leading to acute respiratory distress syndrome (ARDS), life-threatening lung damage, and increased mortality of COVID-19 patients. However, the underlying mechanism of how SARS-CoV-2 infection contributes to NF-κB-mediated inflammatory responses that are expected to determine the outcome of SARS-CoV-2 viral replication and pathogenesis is still largely uncharacterized.

Here we focused on characterizing the regulatory functions of SARS-CoV-2 Nsp14 that are required for efficient viral replication. Nsp14 is a conserved, multifunctional viral factor participating in synthesizing and modifying coronaviral sub-genomic (sg) RNAs (9). Nsp14 possesses a 3’ to 5’ exonuclease activity that excises mismatched base pairs during viral RNA replication (10–12), providing a proofreading function that increases the fidelity of viral RNA synthesis (13, 14). Nsp14 also possesses RNA methyltransferase activity required for guanine-N7 methylation (15). Nsp14-mediated guanine-N7 methylation cooperates with 2’-O RNA methylation mainly catalyzed by Nsp10/16, leading to 5’-capping of newly synthesized sgRNAs (16, 17), which not only prevents degradation by host RNA 5’ exonucleases and recognition by host foreign RNA sensors, such as RIG-I (18) but also increases translation efficiently of host ribosomes to synthesize viral proteins (19, 20). Nsp14 has also been reported to reduce the accumulation of viral double-stranded (ds) RNAs and thus dampen the pathogen-associated molecular pattern (PAMP) mediated antiviral response (21). In addition, Nsp14 is known to facilitate recombination between different viral RNAs to generate new strains (22). Compared to these well-studied viral functions of Nsp14, its regulation of host cellular events is much less investigated. An earlier large-scale proteomic analysis reporting candidate interacting partners for all of the SARS-CoV-2 open reading frames (ORFs) indicated that the host inosine-5’-monophosphate dehydrogenase 2 (IMPDH2) protein is one binding partner of SARS-CoV-2 Nsp14 protein (23). Interestingly, IMPDH2 has been identified to play a role in regulating NF-κB signaling (24). Our new results showed that SARS-CoV-2 Nsp14 activates NF-κB signaling and induces IL-8 upregulation, which indeed requires the interaction of Nsp14 with IMPDH2.

## Results

### SARS-CoV-2 Nsp14 causes activation of NF-κB

**Figure 1:**
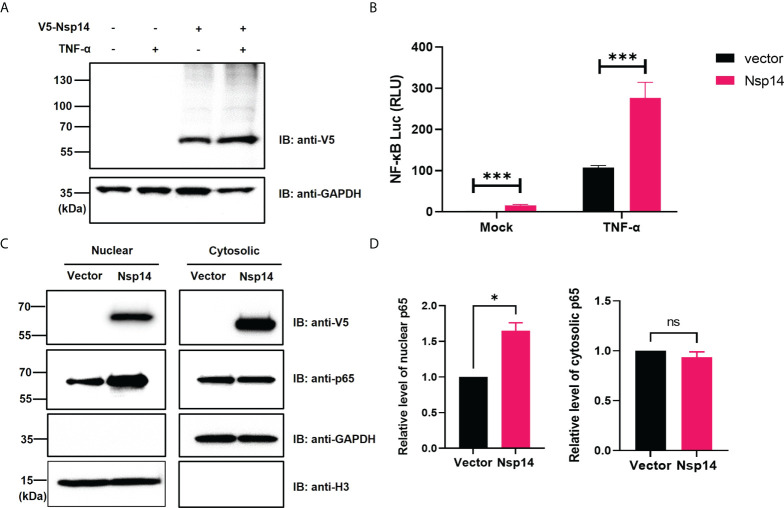
SARS-CoV-2 Nsp14 increases NF-κB activity. **(A–C)** HEK293T cells were transiently transfected with V5-FLAG-Nsp14 or empty vector for 24h and then treated with or without TNF-α for 24h. V5-FLAG-Nsp14 was analyzed by protein immunoblotting **(A)**. HEK293T cells transfected with V5-FLAG-Nsp14 or empty vector along with NF-κB-driven firefly luciferase and TK-driven renilla luciferase reporter vectors were un-treated or treated with TNF-α **(B)**. Luciferase activity (firefly/renilla) in these cells was measured and normalized to the untreated group with the empty vector. HEK293T cells transfected with V5-FLAG-Nsp14 or empty vector were subjected to the nuclear/cytosolic fractionation. V5-FLAG-Nsp14 and NF-κB p65 in the nucleus or cytosol were analyzed by protein immunoblotting **(C)**. Histone H3 was used as the nuclear marker. The intensity of the p65 protein band was quantified and normalized to the empty vector **(D)**. Results were calculated from 3 independent experiments and presented as mean +/- standard error of the mean (SEM). (ns: not significant ; * p<0.05; *** p<0.001; by unpaired Student’s t-test).

We initially investigated the effect of SARS-CoV-2 Nsp14 along with Nsp10 and Nsp16 on certain immune signaling pathways. The pcDNA-V5-FLAG-Nsp14/10/16 vectors were individually transfected in HEK293T, and the expression of the individual proteins was confirmed (**Figure S1A**). We then utilized these expression vectors for interferon-sensitive response element (ISRE) and NF-κB luciferase reporter assays (**Figures S1B, C**). Nsp14 mildly increased ISRE activity at the basal level but caused its decrease in IFN-α-treated HEK293T cells, while Nsp10 and Nsp16 mildly decreased ISRE activity at both conditions, which is consistent with earlier findings (3, 4). On the contrary, only Nsp14 significantly increased NF-κB activity in both untreated and TNF-α-treated HEK293T cells. TNF-α did not affect the expression of transfected Nsp14 in HEK293T cells (**Figure 1A**) but induced a drastic increase of NF-κB activity that was further enhanced by Nsp14 (**Figure 1B**). Thus, we further investigated the Nsp14-induced activation of NF-κB signaling. The impact of Nsp14 on nuclear localization of NF-κB p65 was determined in HEK293T cells transfected with Nsp14. Indeed, Nsp14 expression led to the significant increase of nuclear but not total p65 protein (**Figures 1C, D** and **Figure S2**). These results confirmed that SARS-CoV-2 Nsp14 activates NF-κB signaling.

### SARS-CoV-2 Nsp14 induces upregulation of IL-8

**Figure 2:**
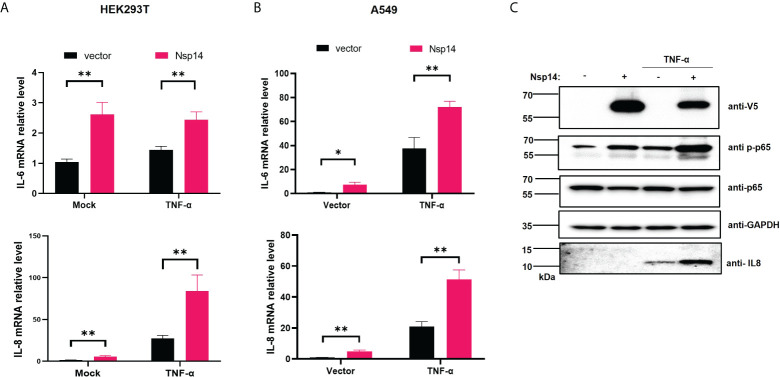
SARS-CoV-2 Nsp14 increases IL-6/8 expression. **(A)** HEK293T cells were transfected with V5-FLAG-Nsp14 or empty vector were un-treated or treated with TNF-α for 24h. The mRNA level of IL-6 and IL-8 in these cells was measured and normalized to the untreated, empty vector-transfected group. **(B)** A549 cells were treated similarly as in **(A)** and analyzed for IL-6 and IL-8 mRNA expression. **(C)** Protein level of Nsp14, phosphate, and total NF-κB p65, as well as IL-8, in A549 cells transfected with or without FLAG-V5-Nsp14 +/- TNF-α was measured by immunoblotting. Results were calculated from at least 3 independent experiments and presented as mean +/- standard error of the mean (SEM). (* p<0.05; ** p<0.01; by unpaired Student’s t-test).

NF-κB plays a critical role in regulating pro-inflammatory gene expression. Since we showed that Nsp14 causes NF-κB activation, we further determined whether Nsp14 induces the expression of IL-6 and IL-8. IL-6 and IL-8 are defined gene targets of NF-κB (25–27). In HEK293T cells transfected with pcDNA-V5-FLAG-Nsp14, IL-6 and IL-8 were consistently and significantly upregulated with or without TNF-α (**Figure 2A**). Results were similar in Nsp14-transfected A549 cells (**Figure 2B**). IL-8 protein was detected in TNF-α treated A549 cells, which further increased due to Nsp14 expression along with the increase of p65 phosphorylation at ser536 (**Figure 2C**). The p65 phosphorylation at ser536 increases p65 nuclear accumulation and NF-κB’s transactivation during inflammation or stress response (28–30). Our results suggested that Nsp14 is capable of increasing NF-κB p65 transactivation to induce IL-8 expression.

**Figure 3:**
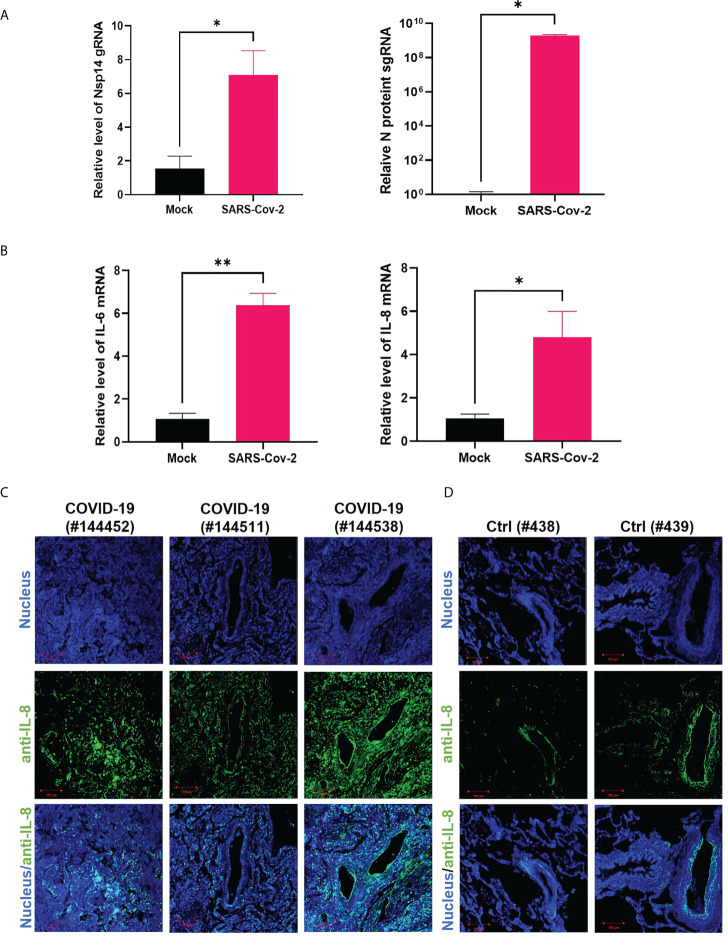
IL-8 is induced by Nsp14 and in SARS-CoV-2 infected lung tissues. **(A, B)** HEK293T-ACE2 cells were infected with wild-type SARS-Cov-2 viruses. Cells were harvested at 24 h. Total RNAs were extracted, and the expression of viral genes (Nsp14, N-protein, **A**) or cytokines (IL-6, IL-8, **B**) was analyzed by RT-qPCR and normalized to mock infection. Results were calculated from 3 technical repeats and presented as mean +/- standard error of the mean (SEM). (* p<0.05; ** p<0.01; by unpaired Student’s t-test). **(C, D)** Dissected lung tissues from COVID19 patients (**C**, donors #144452, #144511, #144538) or non-infected donors (**D**, donors #438, #439) were analyzed for IL-8 expression by immunofluorescence (green). Nuclei were stained with Hoechst (blue). Scale bar: 100 µm.

We next confirmed whether infection of cells with SARS-CoV-2 also induces upregulation of IL-6 and IL-8. HEK293T-ACE2 cells were infected with the SARS-CoV-2 viral strain USA‐WA1/2020 (31). The expression of viral genes, Nsp14 and nucleocapsid (N), was readily detected (**Figure 3A**). The SARS-CoV-2 infection also led to the upregulation of IL-6 and IL-8 (**Figure 3B**). We employed immunofluorescence staining assays to determine whether IL-8 upregulation occurs in lung tissue samples dissected from deceased COVID-19 patients. The results showed that IL-8 expression is consistently higher in COVID-19 patients (**Figure 3C**) compared to un-infected cases (**Figure 3D**). Indeed, earlier studies of deceased samples of COVID-19 patients identified that IL-8 induction occurs in SARS-CoV-2 infection (5, 32). Our results suggested that Nsp14 contributes to IL-8 induction through NF-κB activation. We primarily focused on IL-8 as the target gene of NF-κB for further investigation since it is known that IL-8 has a role in favoring viral infection by inhibiting the antiviral action of IFNα (33).

### IMPDH2 binds to Nsp14 and contributes to Nsp14-mediated induction of IL-8

**Figure 4:**
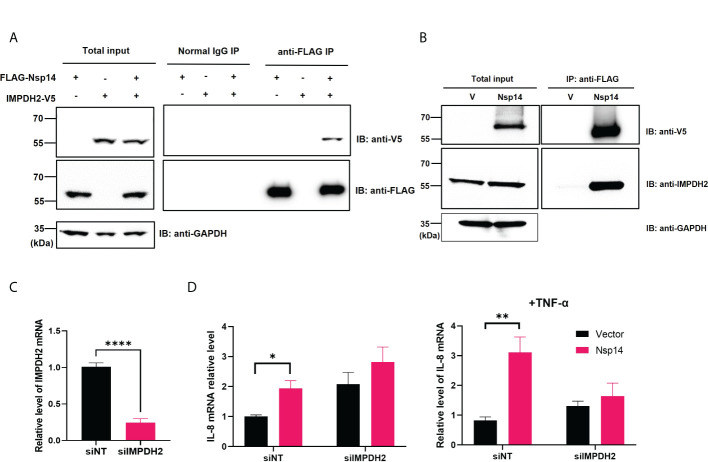
IMPDH2 associates with Nsp14 and is required for IL-8 upregulation by Nsp14. **(A)** HEK293T cells were transiently transfected with the vector expressing FLAG-Nsp14 or V5-IMPDH2, alone or together. Cell lysates were prepared and subjected to protein co-immunoprecipitation (co-IP) assays using anti-FLAG or control IgG antibody. Precipitated protein samples were analyzed by protein immunoblotting using anti-V5 and anti-FLAG antibodies. **(B)** HEK293T cells were transiently transfected with the empty vector **(V)** or FLAG-V5-Nsp14 vector. Cell lysates were prepared and subjected to protein co-IP assays using an anti-FLAG antibody. Precipitated protein samples were analyzed by protein immunoblotting using anti-V5 and anti-IMPDH2 antibodies. **(C)** HEK293T cells were transiently transfected with IMPDH2 or non-targeting (NT) siRNAs. The mRNA level of IMPDH2 was measured and normalized to siNT. **(D)** HEK293T cells transfected with IMPDH2 or NT siRNAs were further transfected with V5-FLAG-Nsp14 or empty vector. These cells were untreated or treated with TNF-α. Total RNAs were extracted. IL-8 mRNA was analyzed and normalized to the siNT and empty vector-transfected group. Results were calculated from 3 independent experiments and presented as mean +/- standard error of the mean (SEM). (*p<0.05; ** p<0.01; **** p<0.0001; by unpaired Student’s t-test).

**Figure 5:**
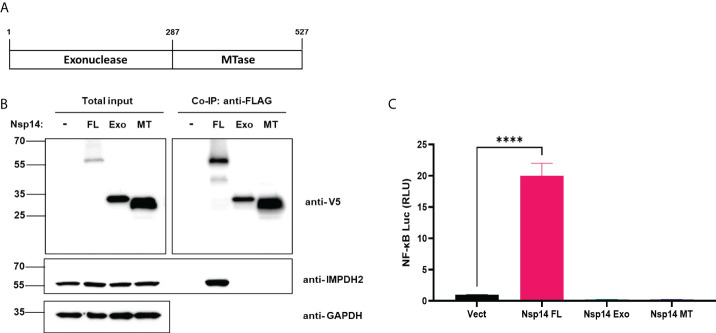
Full-length Nsp14 protein but not truncated domains interact with IMPDH2 and induce NF-κB activation. **(A)** We cloned the truncated Nsp14 protein to encode the exonuclease domain (Exo, aa1-287, MW ~30 kDa) or RNA methyltransferase domain (MT, aa288-527, MW ~27 kDa) with N-terminal FLAG and V5 tags. **(B)** pcDNA vector expressing full-length (FL), Exo, or MT Nsp14 was transfected in HEK293T cells. At 48h post of transfection, cells were harvested. Lysates were prepared and subjected to protein co-IP assays using an anti-FLAG antibody. Precipitated protein samples were analyzed by immunoblotting. **(C)** HEK293T cells were transfected with pcDNA vector expressing full-length (FL), Exo, or MT Nsp14, or empty vector, along with NF-κB-driven firefly luciferase and TK-driven renilla luciferase reporter vectors. At 48h post of transfection, luciferase activity (firefly/renilla) was measured and normalized to the empty vector. Results were calculated from 3 technical repeats and presented as mean +/- standard error of the mean (SEM). (**** p<0.001; by one-way ANOVA and Tukey’s multiple comparison test).

We first confirmed the putative protein interaction of Nsp14 with IMPDH2 (23) by protein co-immunoprecipitation (co-IP) assays in HEK293T cells co-transfected with the pLEX-V5-IMPDH2 and pEZY-FLAG-Nsp14 vectors (**Figure 4A**). We also used the protein co-IP assays to confirm the protein interaction of FLAG-V5-Nsp14 with endogenous IMPDH2 (**Figure 4B**). As the next step, we determined whether endogenous IMPDH2 is required for IL-8 induction by Nsp14. IMPDH2-targeting or non-targeting (NT) siRNAs were transfected in HEK293T cells, and an efficient knockdown of endogenous IMPDH2 was confirmed (**Figure 4C**). Remarkably, IMPDH2 knockdown abolished the IL-8 induction by Nsp14 in HEK293T cells without or with TNF-α (**Figure 4D**). However, overexpression of IMPDH2 had no significant effect on NF-κB activation by Nsp14 in HEK293T cells with or without TNF-α (**Figure S3**). In order to further pinpoint which domain(s) of Nsp14 binding to IMPDH2, we cloned the truncated Nsp14 protein to encode the exonuclease domain (Exo, aa1-287, MW ~30 kDa) or RNA methyltransferase domain (MT, aa288-527, MW ~27 kDa) (9, 13, 34) with N-terminal FLAG and V5 tags (**Figure 5A**). We transfected full-length (FL), Exo, or MT Nsp14 cDNA in HEK293T cells, and NSP14 proteins were readily expressed (**Figure 5B**). In the above HEK293T cells, we determined the interaction of FL or truncated Nsp14 protein with endogenous IMPDH2 protein through co-IP assays. Results showed that only the FL Nsp14 protein binds to endogenous IMPDH2 (**Figure 5B**). Consistently, only the FL Nsp14 protein increased NF-κB-driven luciferase activities (**Figure 5C**). Thus, both functional domains of NSP14 involve in its interaction with IMPDH2 and the induction of NF-κB activation corporately.

### IMPDH2 inhibition blocks Nsp14-mediated NF-κB activation and IL-8 induction

**Figure 6:**
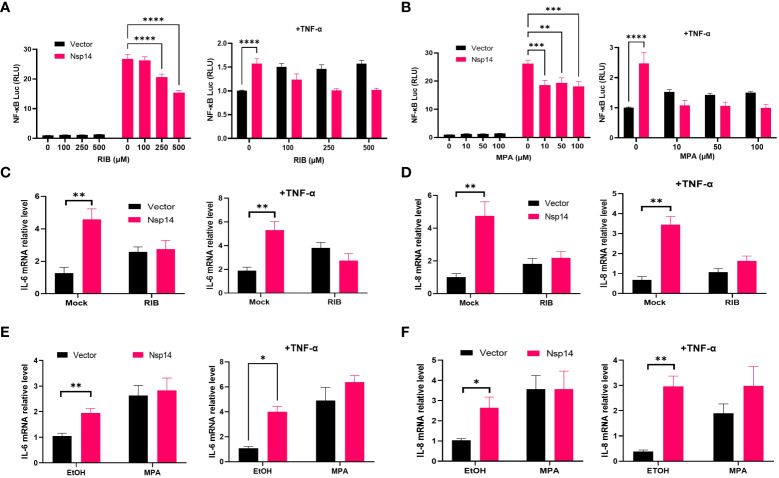
IMPDH2 inhibition reduces Nsp14-mediated NF-κB activation and IL8 induction. **(A)** HEK293T cells transfected with V5-FLAG-Nsp14 or empty vector along with NF-κB-driven firefly luciferase and TK-driven renilla luciferase reporter vectors were treated with ribavirin (RIB) at 24h post of transfection for 24h without or with TNFα stimulation. Luciferase activity (firefly/renilla) in these cells was measured and normalized to that of un-treated, empty vector-transfected cells. **(B)** Mycophenolic acid (MPA) was tested similarly as in **(A)**. Results were calculated from at least 2 independent experiments and presented as mean +/- standard error of the mean (SEM). (** p< 0.01; *** p< 0.001; **** p< 0.0001 by two-way ANOVA and Tukey’s multiple comparison test). **(C, D)** HEK293T cells transfected with V5-FLAG-Nsp14 or empty vector were treated with 500 µM RIB at the basal or TNF-α-stimulated condition. Total RNAs were extracted. IL-6 **(C)** and IL-8 **(D)** mRNA was analyzed and normalized to the mock treatment of the empty vector-transfected group. **(E, F)** 100 µM MPA was tested similarly as in **(C, D)**, and results were normalized to the solvent control (0.1% ethanol, EtOH) of the empty vector-transfected group. Results were calculated from 3 independent experiments and presented as mean +/- standard error of the mean (SEM). (* p<0.05; ** p<0.01; by unpaired Student’s t-test).

Since IMPDH2 is required for IL-8 induction by Nsp14, we expected that its inhibition would reduce Nsp14-mediated NF-κB activation and IL-8 induction. We tested two reported IMPDH2 inhibitors, ribavirin (RIB) and mycophenolic acid (MPA) (23, 35). RIB is a synthetic nucleoside that occupies the IMPDH2 catalytic site to inhibit IMP conversion to xanthosine 5’-phosphate (XMP) during the guanine nucleotide (GTP) biosynthesis (35–37). MPA shares similar features with the IMPDH2 cofactor, nicotinamide adenine dinucleotide (NAD^+^). MPA stacks and traps the XMP intermediate at the catalytic site to inhibit IMPDH2 enzyme activity (35, 38). We confirmed that NF-κB activation by Nsp14 significantly decreases in HEK293T cells treated with RIB (**Figure 6A**) or MPA (**Figure 6B**) at multiple doses in the absence or presence of TNF-α using the NF-κB luciferase reporter assays. Treatment of HEK293T cells with RIB (**Figure S4A**) or MPA (**Figure S4B**) at the similar doses caused no obvious cytotoxicity through cell viability assays. Likewise, treatment of HEK293T cells with RIB (**Figures 6C, D**) or MPA (**Figures 6E-F**) also caused the reduction of both IL-6 and IL-8 mRNA induction by Nsp14. Furthermore, we treated Nsp14-transfected A549 cells with RIB or MPA, which decreased the TNF-a induced p65 phosphorylation and IL-8 protein expression (**Figure S4C**). These results supported the model that IMPDH2 is required for Nsp14’s function to activate NF-κB and induce IL-8. However, these IMPDH2 inhibitors had no effect on disrupting the Nsp14-IMPDH2 protein interaction measured by co-IP assays (**Figure S4D**).

### IMPDH2 or NF-κB inhibition restricts viral infection of SARS-CoV-2 in cell culture

**Figure 7:**
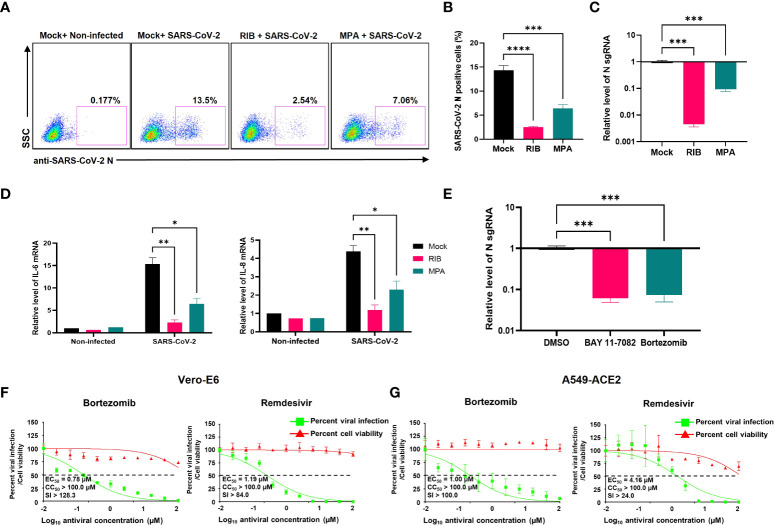
IMPDH2 and NF-κB inhibitors restrict the SARS-CoV-2 infection in cell culture. **(A-D)** A549-ACE2 cells were treated with RIB (500 μM), MPA (100 μM), or mock, and simultaneously infected with SARS-Cov-2 viruses for 24 h. The SARS-CoV-2 infection was detected by intracellular staining of SARS-CoV-2 N protein **(A)**. The percentage of SARS-CoV-2 N protein-positive cells was calculated **(B)**. Cells were harvested for RNA extraction, and N protein sgRNA was analyzed and normalized to the mock treatment **(C)**. The mRNA of IL-6 and IL-8 was analyzed and normalized to the non-infected cell with the mock treatment **(D)**. **(E)** HEK293T-ACE2 cells were infected with SARS-CoV-2 in the presence of BAY 11-7082 (10 µM), bortezomib (10 µM), or DMSO solvent control for 24 h. The sgRNA level of N protein was analyzed and normalized to DMSO. Results were calculated from 3 technical repeats and presented as mean +/- standard error of the mean (SEM). (* p<0.05; ** p<0.01; *** p<0.001; **** p<0.001; by one-way **(B, C, E)** or two-way ANOVA **(D)** and Tukey’s multiple comparison test). **(F-G)** Vero E6 **(F)** or A549-ACE2 **(G)** cells were briefly infected with SARS-CoV-2, followed by treatment of indicated compounds (bortezomib, remdesivir). At 24 hpi, the above cells were subjected to PRMNT assay at four biological replicates. Results were calculated as mean +/- standard deviation (SD). The dotted line indicates the 50% inhibition. The selectivity index (SI) is presented as CC_50_/EC_50_.

We next tested whether IMPDH2 inhibitors (RIB, MPA) impact SARS-CoV-2 infection *in vitro*, considering that virus-mediated NF-κB activation modulates viral infection (39–42). Indeed, we showed that the infection rate of SARS-CoV-2 decreases in both A549-ACE2 and HEK293T-ACE2 cells treated with RIB or MPA through quantification of cells expressing N protein by immunofluorescence staining assays (**Figures 7A, B**, **S5A, B**) or sgRNA level by RT-qPCR (**Figures 7C**, **S5C**). Consistently, we also identified that treatment of RIB or MPA leads to a significant reduction of IL-6 and IL-8 expression (**Figure 7D**). The results of IMPDH2 inhibitors also aligned with those of NF-κB inhibitors. Treatment of HEK293T-ACE2 cells with the NF-κB inhibitors, including BAY 11-7082 (43) and bortezomib (44), significantly reduced the sgRNA level of SARS-CoV-2 N protein measured by RT-qPCR (**Figure 7E**). In order to confirm the antiviral activity of NF-κB inhibitors, we further performed the plaque reduction microneutralization (PRMNT) and cell-viability assays with the serial dilution of these drugs in Vero E6 and A549-hACE2 cells (**Figures 7F, G**). Bortezomib potently blocked SARS-CoV-2 infection in both cells without obvious cytotoxicity, comparable to remdesivir. However, the anti-SARS-CoV-2 activity of BAY 11-7082 correlated with the increased cytotoxicity in both cells (**Figure S5D**). These NF-κB inhibitors also had no effect on disrupting the Nsp14-IMPDH2 protein interaction (**Figure S4E**).

## Discussion

Besides the well-known viral functions of SARS-CoV-2 Nsp14 to control modification and replication of viral RNA genomes, earlier studies illustrated that Nsp14 suppresses Type 1 IFN signaling and nuclear translocation of IRF3 to facilitate viral invasion of the host’s antiviral immune response (3, 4). Our results showed that Nsp14, which is expressed at the early stage of primary infection (7), also affects other cell signaling pathways, such as NF-κB signaling (**Figure 1**), likely to support viral replication. Activation of NF-κB may further trigger the production of downstream pro-inflammatory cytokines to initiate the cytokine storm and contribute to ARDS. In this study, we identified that Nsp14 increases nuclear translocation of p65 and induces the expression of NF-κB’s downstream cytokines, such as IL-6 and IL-8, which have also been detected in lung tissues of COVID-19 patients (5, 32) and animal models of SARS-CoV-2 infection (7). These cytokines are reported to play a critical role in regulating the recruitment and infiltration of immune cells (macrophages, neutrophils) during viral infection (43, 44). Infiltrating immune cells may further escalate inflammatory responses leading to lung damage. Indeed, we showed that IL-8 expression is much higher in lung tissue samples of COVID-19 patients than in uninfected controls (**Figures 3C, D**). We identified that only the FL Nsp14 protein binds to endogenous IMPDH2 protein (**Figure 5B**) and induces NF-κB activation (**Figure 5C**). These results align with other findings reporting that Nsp14 needs both its exonuclease and methyltransferase domains to shut down the host’s protein translation (45). However, due to current technical limitations one remaining question is whether Nsp14 protein expressed from SARS-CoV-2 viral genome truly contributes to NF-κB activation and IL-6/8 induction, which needs future confirmation. A recent study showed that Nsp14 interacts with SIRT1/SIRT5 to decrease NRF2/HMOX1 signaling while increase oxidant stress and inflammatory responses (46). Nsp14 H268A mutant and other exoribonuclease-deficient mutants still inhibit NRF2/ARE-driven transcription, suggesting that Nsp14 may affect cellular signaling *via* protein-protein interaction independent of its exoribonuclease activity. However, it is intriguing to test whether Nsp14 exoribonuclease-deficient mutants (such as H268A) have an impact on Nsp14’s function to induce NF-κB signaling, since our results indeed showed that exoribonuclease domain of Nsp14 is required for NF-κB activation (**Figure 5C**).

**Figure 8:**
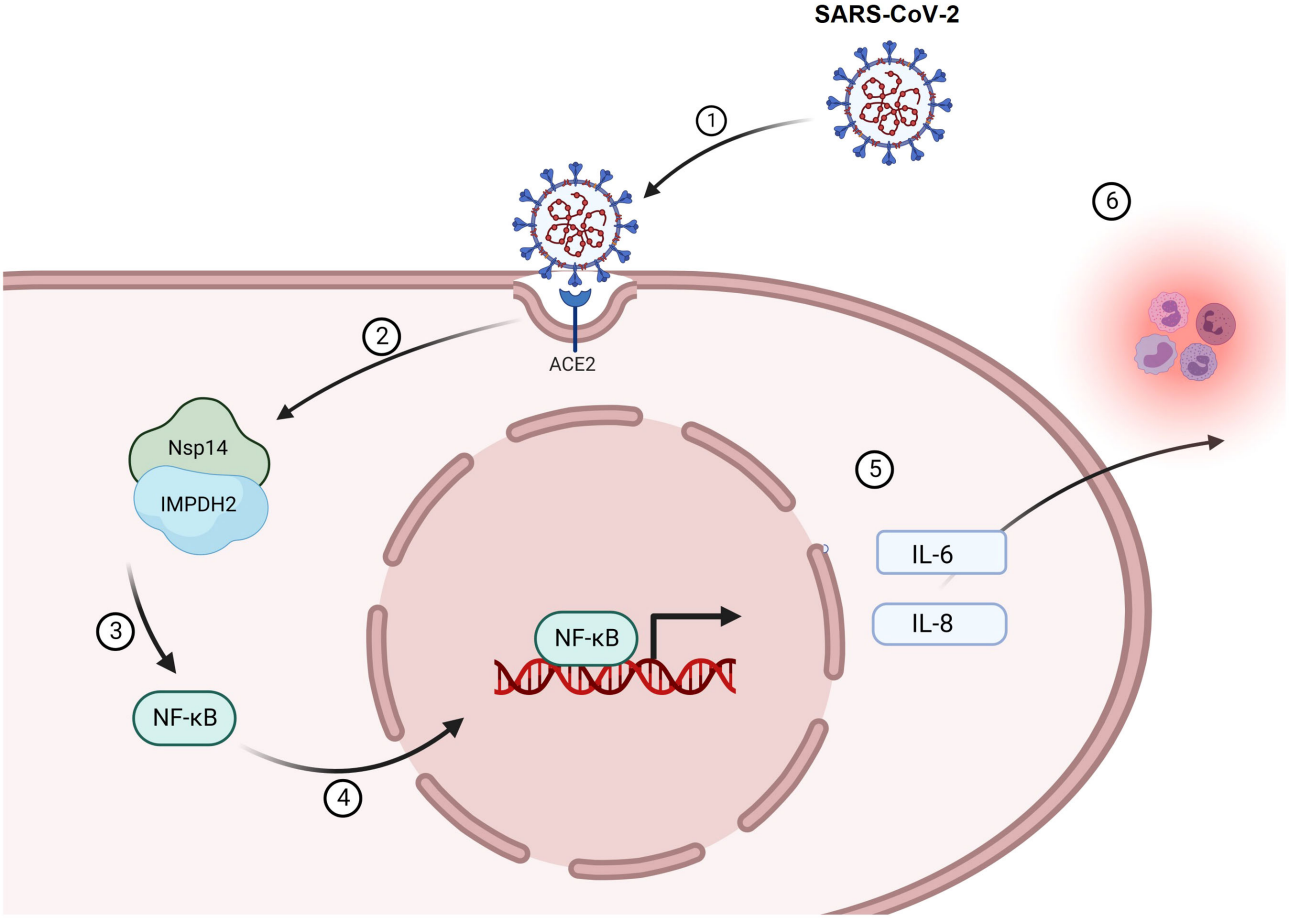
A working model of Nsp14-mediated NF-κB activation during SARS-CoV-2 infection. Infection of SARS-CoV-2 (1) leads to the expression of Nsp14 (2) that interacts with IMPDH2 (3). Such interaction promotes the nuclear translocation of NF-κb p65 (4) and its activation, which upregulates the expression of downstream cytokines, including IL-6 and IL-8 (5). Expression of IL-6 and IL-8 may further amplify the inflammatory response from immune cells contributing to viral pathology in COVID (6) and also, in return, benefit SARS-CoV-2 infection.

Another key finding is that IMPDH2 is a host mediator of Nsp14 involved in NF-κB activation, verified by both genetic knockdown (**Figure 4**) and chemical inhibition (**Figure 6**). We confirmed the protein interaction of Nsp14 with IMPDH2, which was initially reported in earlier proteomic studies (23, 32). Previous results also suggested that IMPDH2 benefits the budding of Junín mammarenavirus (JUNV), propagation of lymphocytic choriomeningitis virus (LCMV) (47), and replication of human norovirus (HuNV) (48). IMPDH2 inhibitors have been used for treating hepatitis C virus (HCV) (35, 49). Our results suggested that IMPDH2 likely supports the SARS-CoV-2 infection and Nsp14-mediated NF-κB activation as well. IMPDH2 is a protein target of certain immunosuppressive drugs used for organ transplantation and allograft rejection (38, 50, 51), and it has been reported to regulate NF-κB signaling (24, 52). In an earlier study, RIB (the IMPDH2 inhibitor) decreased the IL-6/IL-8 secretion in the animal models of rotavirus infection (53). Another study also showed that MPA (the IMPDH2 inhibitor) decreased the NF-κB activation and induction of IL-8 (54) and IL-6 (55, 56). Nsp14 may hijack IMPDH2 for NF-κB activation (24), contributing to abnormal inflammatory responses. IL-6 from infected cells may stimulate macrophages, pathological fibroblasts, Th2 and Th17 cells, and initiate inflammatory or immunopathological responses that dysregulate extracellular matrix, impair tissue repairing, and facilitate tissue injury (57–61). IL-8 may attract neutrophils, stimulate granulocytes’ response to tissue damage, and generate ARDS-related micro thrombosis (62, 63). IL-8 induction could initiate a positive feedback *via* autocrine of attracted neutrophils (63), which may also support viral replication of SARS-CoV-2 (64) and inhibit SARS-CoV-2 specific T-cell responses (65). In terms of possible molecular mechanisms, since IMPDH2 participates in regulating the host nucleotide metabolism (66, 67), it may further modulate cellular stress response and downstream NF-κB activation (67–69). This metabolism disruption caused by Nsp14 might increase the phosphorylation of IKKβ and IκBα to further promote nuclear translocation and phosphorylation of p65 (24). Future studies will be needed to understand further how Nsp14 and IMPDH2 cooperate to activate NF-κB. In addition, we also noticed that Nsp14 partially localizes in the nuclei of cells (**Figures 1C, D** **)**, similar to findings from other groups (70, 71). Thus, Nsp14 may encode other cellular functions. Nsp14 may associate with and modify the host cellular RNAs *via* its exonuclease and methyltransferase activities. Nsp14 may also affect the transcriptional activity of nuclear p65 and the expression of its gene targets. Our study has translational significance since we showed that both IMPDH2 inhibitors, RIB and MPA, effectively reduce viral replication of SARS-CoV-2 and expression of NF-κB’s downstream cytokines (IL-8 and IL-6) induced by SARS-CoV-2 (**Figures 7A–D**). The antiviral effect of IMPDH2 inhibitors is likely through inhibition of NF-κB, supported by our results showing that NF-κB inhibitors, particularly bortezomib, indeed restrict viral infection of SARS-CoV-2 in cell culture as well (**Figures 7E–G**). It has been reported that IL-8 increases the replication of human immunodeficiency virus-1 (HIV-1), HCV, and cytomegalovirus (CMV) (72–75). SARS-CoV-2 Nsp14 induces the NF-κB signaling and downstream cytokines, which may support the host cell proliferation and survival, or prevent cell apoptosis, thus benefiting viral replication (42, 76). As the supportive evidence, a recent study showed that knockdown of NF-κB p50 or IL-8 indeed impairs SARS-CoV-2 viral RNA expression and its replication in A549-ACE2 cells (64). RIB and MPA are both FDA-approved drugs for treating HCV infection and transplant organ rejection, respectively. Our findings are supported by recent results showcasing the therapeutic potential of RIB and MPA for treating COVID-19 and SARS-CoV-2 infection. The combination of RIB with IFN β-1b and Lopinavir–Ritonavir therapy is currently in clinical trials for treating SARS-CoV-2 infection (77), which has been shown to significantly alleviate the COVID-19 symptoms and suppress IL-6 levels in serum. In another preclinical study, MPA was reported to inhibit SARS-CoV-2 replication (78) and viral entry (79). In addition, bortezomib is an FDA-approved antineoplastic agent and would be promising to treat SARS-CoV-2, which will be further investigated. Overall, our study delineated a potentially new mode of action (MOA) for these IMPDH2 inhibitors, which may disrupt the Nsp14-IMPDH2 axis that plays a crucial role in regulating activation of NF-κB signaling and induction of its downstream cytokines (**Figure 8**).

## Material and methods

### Cell culture

HEK293T cells (Cat. # CRL-3216, ATCC) were cultured in Dulbecco’s modified Eagle’s medium (DMEM, Cat # D5796, Sigma). A549 cells (Cat. # CCL-185, ATCC) were cultured in F12K medium (Cat. # 21127030, Gibco™). Vero E6 cells (Cat. # CRL-1586, ATCC) were cultured in DMEM. HEK293T cells stably expressing ACE2-GFP were previously described (31). A549-ACE2 cells were obtained through BEI Resources, NIH NIAID (Cat # NR53821). Cell culture medium contained 10% fetal bovine serum (FBS, Cat. # 10437028, Thermo Fisher), penicillin (100 U/ml)/streptomycin (100 μg/ml) (Cat. # MT30002CI, Corning).

### Compounds and antibodies

Recombinant human TNF-α (Cat. # 554618) was purchased from BD. Biosciences. Ribavirin (RIB, Cat. # R0077) was purchased from Tokyo Chemical Industry (TCI). Mycophenolic acid (MPA, Cat. # M3546), BAY 11-7082 (Cat. #B5556-10MG), and bortezomib (Cat. # 5043140001) were purchased from Sigma-Aldrich. Remdesivir was purchased from AOBIOUS.

Anti-V5 (Cat. # R960-25), HRP-conjugated anti-V5, and goat HRP-conjugated anti-mouse IgG (H+L) secondary antibody (Cat. # 31430) were purchased from Thermo Fisher Scientific. Anti-GAPDH antibody (Cat. # sc-32233) was purchased from Santa Cruz Biotechnology. Anti-NF-κB p65 (Cat. #8242), anti NF-κB p65 ser536 phosphorylation (Cat. #3033), anti-FLAG (Cat. # 2368), anti-H3 (Cat. # 9715S), and goat HRP-conjugated anti-rabbit IgG (Cat. # 7074) antibodies were purchased from Cell Signaling Technology. Anti-IL8 antibody (Cat. # 554717) was purchased from BD. Biosciences. The anti-IMPDH2 antibody (Cat. # 12948-1-AP) was purchased from Proteintech Group. The anti-SARS-CoV-1/2 NP 1C7C7 antibody was purchased from Sigma-Aldrich.

### Plasmids

pLEX-IMPDH2-V5 vector was picked from the MISSION TRC3 human LentiORF library from Sigma-Aldrich. The pcDNA-FLAG-V5-Nsp10/14/16 vectors were constructed from pDONR223 SARS-CoV-2 Nsp10 (Cat. # 141264, Addgene), Nsp14 (Cat. # 141267, Addgene), and Nsp16 (Cat. # 141269, Addgene) vectors to the pcDNA3.1-3xFLAG-V5-ccdB (Cat. # 87064, Addgene) destination vector using Gateway™ LR Clonase™ II Enzyme Mix (Cat. # 11791020, Invitrogen). pEZY-FLAG-Nsp14 vector was constructed from pDONR223 SARS-CoV-2 Nsp14 vector to the pEZY-FLAG (Cat # 18700, Addgene) destined vector. pcDNA-FLAG-V5-Nsp14 Exo/MT vectors were constructed. Nsp14 Exo/MT domain was PCR amplified with the Phusion Flash High-Fidelity PCR Master Mix (Cat. #F548S, Thermo Scientific) using the following primers. Exo forward: 5’-GGGGACAAGTTTGTACAAAAAAGCAGGCTGCATGGCTGAGAACGTGACCG-3’; Exo reverse: 5’-GGGGACCACTTTGTACAAGAAAGCTGGGTATTACACGAAGCACTCGTGCAC-3’;MT forward: 5’-GGGGACAAGTTTGTACAAAAAAGCAGGCTGCATGAAGCGTGTGGACTGGACC-3’; MT reverse: 5’-GGGGACCACTTTGTACAAGAAAGCTGGGTATTACTGCAGCCTGGTGAAGGTG-3’.

TG-3’. The PCR products of Nsp14 domains were recombined in the pDONR223 vector *via* B/P cloning using the Gateway™ BP Clonase™ II Enzyme mix (Cat. # 11789020, Invitrogen), and subsequently in pcDNA3.1-3xFLAG-V5-ccdB vector *via* L/R cloning. The pLEX-FLAG-V5 vector was constructed by cloning the FLAG sequence to the pLEX-307 (Cat # 41392, Addgene) vector. The pNF-κB-luciferase vector (PRDII_4_–luc in the pGL3 vector) was the gift from Dr. Jacob Yount’s lab (80). The pIRES-luciferase vector (Cat. # 219092) was acquired from Agilent Technologies. The pRL-TK Renilla Luciferase vector (Cat. # AF025846) was purchased from Promega.

### Transient transfection

For Nsp14 overexpression, we performed the transient transfection in HEK293T or A549 cells using TurboFect transfection reagents (Cat. # R0531, Thermo Scientific). Briefly, cells were seeded and incubated with the mixture of plasmids with Turbofect for 24 h. The medium was changed, followed by treatment of TNF-α or compounds. For IMPDH2 knockdown, 20 nM siRNA (IMPDH2 assay ID: s7417, sense: 5’-CCAAGAAAAUCACUCUUtt-3’, Ambion by Life technologies; non-targeting control: Silencer™ Negative Control No. 4 siRNA, si NT, Cat. # AM4641, Invitrogen) was reversely transfected in HEK293T cells using Lipofectamine™ RNAiMAX Transfection Reagent (Cat. # 13778030, Invitrogen). Cells were kept in culture for 48h and subjected to qPCR analysis to measure gene expression.

### Protein immunoblotting

Protein immunoblotting was performed following our previously published protocols (81, 82). Briefly, cells were harvested, washed by PBS, and pelleted. Cell pellets were lysed in RIPA buffer (Cat. #20-188, Millipore) containing protease inhibitor cocktail (Cat. # A32965, Thermo Scientific) on ice, followed by brief sonication to prepare cell lysate. The BCA assay kit (Cat. #23225, Thermo Scientific) was used to quantify the total protein amount in cell lysate, which was boiled in the SDS loading buffer with 5% β-mercaptoethanol (Cat. #60-24-2, Acros Organics). The denatured protein samples were separated by Novex™ WedgeWell™ 4-20% SDS-PAGE Tris-Glycine gel and transferred to PVDF membrane (iBlot™ 2 Transfer Stacks, Invitrogen) using iBlot 2 Dry Blotting System (Cat. # IB21001, Thermo Scientific). The membranes were blocked by 5% milk in PBST and probed by the specific primary antibodies at 4°C overnight, followed by the HRP-conjugated secondary antibodies. The membranes were developed using the Clarity Max ECL substrate (Cat. # 1705062, Bio-Rad).

### Luciferase reporter assays

HEK293T cells were transfected with ISRE or NF-κB luciferase vector along with pRL-TK renilla luciferase vector with or without the indicated vector expressing Nsp14. At 24 h post of transfection, the medium was changed, and cells were treated with 10 ng/ml TNF-α or un-treated for 24h. Cells were lysed using the Dual-Glo^®^ Luciferase Assay System (Cat. #E2920, Promega). Luciferase/renilla signal intensity was detected using Biotek Cytation5 and analyzed by GEN5 software (Biotek). Cell viability assays were performed for HEK293T cells treated with inhibitors for 24h by using CellTiter-Glo^®^ (Cat. # G7571, Promega), and the results were normalized to the solvent control.

### Nuclear and cytoplasmic extraction

HEK293T cells were transfected by pcDNA-FLAG-V5-Nsp14 or control vector pLex307-FLAG-V5 for 24h and changed to fresh completed DMEM medium for further 24 h culture. Cells were collected, washed twice with 1× PBS, and subjected to the nucleus and cytoplasm extraction using NE-PER Nuclear and Cytoplasmic Extraction Reagents (Cat. #78833, Thermo Scientific) following the manufacturer’s instructions and our previous studies (81). Total proteins in the whole-cell lysates from the same number of cells were extracted using 1× RIPA buffer. Extractions from nuclear, cytoplasmic proteins and the total cell lysate proteins were denatured and boiled with 4× LDS sample buffer (Cat. #NP0007, Invitrogen) and subjected to immunoblotting analysis with equal protein loading of extracts (~20 µg/lane). Anti-GAPDH and anti-histone H3 immunoblotting were used as internal controls to determine the cytoplasmic and nuclear fractions.

### Protein co-immunoprecipitation

Protein co-IP assays were performed following the previously published protocol (81). Briefly, protein A/G magnetic beads (Cat. # 88802, Thermo Scientific) and anti-FLAG M2 magnetic beads (Cat. # M8823, Sigma-Aldrich) were washed with 1× RIPA buffer containing protease inhibitor cocktail. Cellular lysates were precleared with the empty magnetic beads for 1 h at 4°C on a 360° tube rocker. The cell lysate was incubated with anti-FLAG M2 magnetic beads for pull-down of FLAG-Nsp14 protein at 4°C overnight with constant rotation. Protein immunocomplexes were washed by RIPA buffer and boiled in SDS loading buffer containing 5% 2-mercaptoethanol, followed by protein immunoblotting. A normal mouse IgG antibody (Cat. # sc-2025, Santa Cruz) was used as the control in parallel.

### Quantitative reverse transcription PCR

RT-qPCR assays were performed following the previously published protocol (83). Total RNAs from harvested cells were extracted using the NucleoSpin RNA extraction kit (Cat. # 740955.250, MACHEREY-NAGEL), and 0.4-1 μg RNA was reversely transcribed using the iScript™ cDNA Synthesis Kit (Cat. # 1708890, Bio-Rad). Real-time qPCR was conducted using the iTaq™ Universal SYBR^®^ GreenSupermix (Cat. # 1727125, Bio-Rad). The PCR reaction was performed on a Bio-Rad CFX connect qPCR machine under the following conditions: 95°C for 10 m, 50 cycles of 95°C for 15 s, and 60°C for 1 m. Relative gene expression was normalized to GAPDH internal control as the 2^-ΔΔCt^ method: 2 ^(ΔCT of targeted gene - ΔCT of GAPDH)^. The following primers were used. IL-4 forward: 5’-GTTCTACAGCCACCATGAGAA-3’, reverse: 5’-CCGTTTCAGGAATCAGATCA-3’; IL-6 forward: 5’-ACTCACCTCTTCAGAACGAATTG-3’, reverse: 5’-CCATCTTTGGAAGGTTCAGGT-TG-3’ (61); IL-8 forward: 5’-CTTGGCAGCCTTCCTGATTT-3’; reverse: 5’-GGGTGGAAAGGTTT-GGAGTATG-3’; Nsp14 forward: 5'-ACATGGCTTTGAGTTGACATCT-3',reverse: 5'-AGCAGTGGAAAAGCATGTGG-3' IMPDH2 forward: 5′- CTCCCTGGGTACATCGACTT-3′, reverse: 5′-GCCTCTGTGACTGTGTCCAT-3′ (83); GAPDH forward: 5′-GCCTCTTGTCTCTTAGATTTGG-TC-3′, reverse: 5′-TAGCACTCACCATGTAGTTGAGGT-3′.

SARS-CoV-2-TRS-L (N sgRNA forward): 5′-CTCTTGTAGATCTGTTCTCTAAACGAAC-3′,

SARS-CoV-2-TRS-N (N sgRNA reverse): 5′-GGTCCACCAAACGTAATGCG-3′ (84)

### Viral infection

SARS-CoV-2 strain USA-WA1/2020 was obtained from BEI Resources, NIH, NIAHD (Cat # NR52281) and was plaque purified in Vero E6 cells to identify plaques lacking furin cleavage site mutations. A WT virus plaque was then propagated on Vero E6 cells stably expressing TMPRSS2 (kindly provided by Dr. Shan-Lu Liu, Ohio State University) for 72 h. The virus was aliquoted, flash-frozen in liquid nitrogen, and stored at -80C. The virus stock was titered on Vero E6 cells by TCID50 assay. For infection assays, the SARS-CoV-2 virus (MOI: 1.0) was added to cells along with drug treatment for 24 h at a BSL3 laboratory of OSU Medical Center. Cells were collected by trypsinization, and either lysed with Trizol reagent (Cat # 15596026, Thermo Scientific) for RNA extraction following the manufacturer’s protocol or fixed with 4% paraformaldehyde in PBS for 1 h prior to staining for flow cytometry. Staining was performed with the anti-SARS-CoV-2 nucleocapsid protein (N) antibody (Cat # 40143-MM08, Sino Biological) as described previously (31, 85). Flow cytometry was performed on a FACSCanto II machine (BD Biosciences). Data were analyzed using FlowJo software.

### Human subjects

The lung specimens from deceased COVID-19 patients were obtained from Biobank at Columbia University Irving Medical Center. The control normal lung specimens were the gifts from Jahar Bhattacharya (Columbia University, NY, USA). For paraffin sections, the lungs were fixed with 4% paraformaldehyde (PFA) at 4°C overnight, dehydrated through a series of grade ethanol, and incubated with Histo-Clear (Cat.5989-27-5, National Diagnostics, USA) at room temperature for 2 hours prior to paraffin embedding. 7 μm thick sections were then prepared from the paraffin blocks and mounted on the slides for staining.

### Protein immunofluorescence

Paraffin-embedded lung tissue blocks were baked on the hotplate at 75°C for 20 min and deparaffinized in xylene. The slides were rehydrated from 100%, 90%, to 70% ethanol and then to PBS. We performed the antigen unmasking using the retriever (Cat. # 62700-10, Electron Microscopy Sciences) with R-Buffer A pH 6.0 (Cat. # 62706-10, Electron Microscopy Sciences) for 2 h to complete the cycle and cool down. Slides were blocked with 20% normal goat serum (NGS) in PBST for 2 h at room temperature. Slides were incubated with an anti-IL-8 antibody (Cat. # 550419, BD Pharmingen™) in 5% NGS with PBS at 4°C overnight. Slides were washed with PBST and incubated with Alexa 488 coated goat anti-mouse antibody in 5% NGS/PBS for 2 h at room temperature. Slides were washed with PBST and stained with Hoechst (1:5000 in PBS, Invitrogen). Coverslips were mounted on slides using ProLong Glass Antifade Mountant (Cat. # P36982, Invitrogen) and dried out in the dark overnight. Confocal images were acquired using the ZEISS LSM 700 Upright laser scanning confocal microscope and ZEN imaging software (ZEISS).

### Plaque reduction microneutralization

PRMNT assay was performed to evaluate the antiviral activity of drugs against SARS-CoV-2 as previously described (86). In brief, Vero-E6 and A549-ACE2 cells were seeded on 96-well plates with 4×10^4^ cells/well in 96-well plates (for quadruplicates) at 24 h prior to viral infection. Cells were inoculated with SARS-CoV-2 (USA-WA1/2020 strain) viruses (100 plaque-forming units (PFU)/well) at 37°C for 1 h in the CO_2_ incubator. After 1 h of viral adsorption, infection media was replaced with post-infection media containing 1% Avicel and 2-fold dilutions of the indicated compounds (starting concentration 100 µM), remdesivir (positive controls), or 0.1% DMSO (negative control), and incubated at 37°C for 24 h. At 24 h post-infection, cells were fixed with 10% neutral formalin for 24 h. Cells were permeabilized with 0.5% Triton X-100 in PBS at room temperature for 15 min and blocked with 2.5% BSA in PBS at 37°C for 1 h. Cells were stained with anti-SARS-CoV nucleocapsid (N) protein monoclonal antibody (1C7C7) in 1% BSA–PBS at 37°C for 1 h. After incubation with the primary monoclonal antibody, cells were washed with PBS and incubated with a secondary peroxidase-conjugated goat anti-mouse IgG (Dako; 1:200) in 1% BSA-PBS for 1 h at 37°C. Following the manufacturer’s instructions, the labeled cells were detected by using the VECTASTAIN^®^ ABC-HRP Kit (Vector Laboratories). Viral plaques were quantified using a CTL ImmunoSpot plate reader and counting software (Cellular Technology Limited). Infection of wild-type SARS-CoV-2 was carried out at a BSL3 laboratory of Texas Biomedical Research Institute. The percentage of viral infection was calculated as below:


$$\text{Viral infection}=\frac{\text{(Number of plaques with drug treatment }-\text{Number of plaques with “No virus”)}}{\text{(Number of plaques with “No drug” ;}-\text{Number of plaques with “No virus”)}}$$

### MTT cell viability assay

The viability of Vero and A549-ACE2 cells was determined using the MTT assay (CellTiter 96 Non-Radioactive Cell Proliferation assay, Promega) following the manufacturer’s instructions and as described previously (87). Briefly, confluent monolayers (96-well plate format, 4×10^4^ cells/well, quadruplicates) of Vero and A549-ACE2 cells were treated with 100 µl of DMEM containing serially diluted (2-fold dilutions, starting concentration of 100 µM) compounds, or 0.1% DMSO (negative control). Plates were incubated at 37°C in a 5% CO_2_ atmosphere for 24 h. Cells and supernatants were treated with 15 µl of Dye Solution and incubated at 37°C in a 5% CO_2_ atmosphere for 4 h. Then, cells were treated with 100 µl of Solubilization Solution/Stop Mix, and absorbance at 570 nm was measured using a Vmax kinetic microplate reader (BioTek). The viability of compound-treated cells was calculated as a percentage relative to values obtained with Vehicle-treated cells (0.1% DMSO). Non-linear regression curves and the median cytotoxic concentration (CC_50_) were calculated using GraphPad Prism software version 8.0.

### Statistics

Statistical analysis was performed using the GraphPad PRISM. Data are presented as mean ± SEM of biological repeats from at least 2 independent experiments. * p<0.05, ** p<0.01, *** p<0.001, or **** p<0.001 indicated the significant difference analyzed by ANOVA or Student’s t-test.

## Data availability statement

The original contributions presented in the study are included in the article/**Supplementary Material**. Further inquiries can be directed to the corresponding author.

## Author contributions

JZ and T-WL conceived and designed this study; T-WL performed most of the experiments; AK performed the SARS-CoV-2 infection and its data processing; J-GP performed the PRMNT assay and its data processing; HL prepared the tissue samples of human subjects; T-WL, NS, and JZ analyzed the results; J-GP, AK, HL, GF, DZ, AB, JQ, LM-S, and JY contributed materials and provided advice for this study. T-WL and JZ wrote the manuscript; JZ supervised the entire study. All authors contributed to the article and approved the submitted version.

## Funding

This study was funded by NIH research grants R01AI150448, R01DE025447, R56AI157872, and R33AI116180 to JZ, and R03DE029716, R01CA260690 to NS.

## Acknowledgments

The authors thank Dr. Mark Peeples (Nationwide Children’s Hospital) and Dr. Jianrong Li (The Ohio State University) for kindly providing plaque purified SARS-CoV-2 for viral propagation. We thank Dr. Sheng-Ce Tao (Shanghai Jiao Tong University) for providing the Nsp14 cloning plasmid. We also thank Dr. Karin Musier-Forsyth and Dr. Shan-Lu Liu at The Ohio State University for their advice on our studies.

## Conflict of interest

J-GP and LM-S are listed as inventors on a pending patent application describing the SARS-CoV-2 antibody 1207B4.

The remaining authors declare that the research was conducted in the absence of any commercial or financial relationships that could be construed as a potential conflict of interest.

## Publisher’s note

All claims expressed in this article are solely those of the authors and do not necessarily represent those of their affiliated organizations, or those of the publisher, the editors and the reviewers. Any product that may be evaluated in this article, or claim that may be made by its manufacturer, is not guaranteed or endorsed by the publisher.
