# Supplementary material for: SARS-CoV-2 Nsp14 protein associates with IMPDH2 and activates NF-κB signaling
Source: Front Immunol. 2022 Sep 13;13:1007089. doi: 10.3389/fimmu.2022.1007089 (PMC9513374; doi:10.3389/fimmu.2022.1007089)
Supplement: Supplementary file 1 [file Presentation_1.pdf]

Fig S1

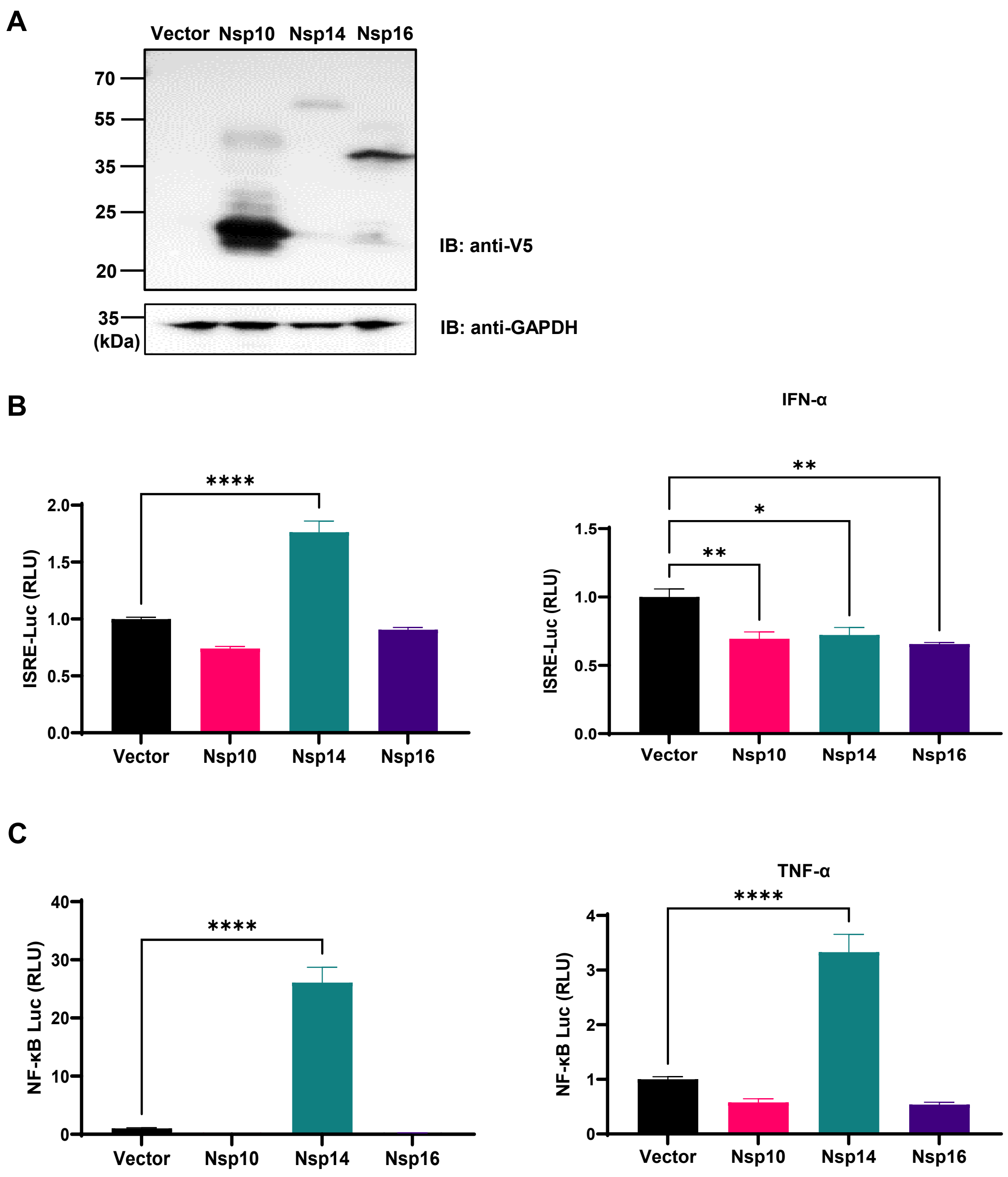
A
Vector
Nsp10
Nsp14
Nsp16
70
55
35
25
20
IB: anti-V5
35
(kDa)
IB: anti-GAPDH
B
ISRE-Luc (RLU)
****
IFN-α
**
*
**
C
NF-κB Luc (RLU)
****
TNF-α
****

Fig S1. (A) HEK293T cells were transiently transfected with the vector expressing V5-FLAG-Nsp10, 14, 16, or empty vector. Protein expression was analyzed by protein immunoblotting. **(B)** HEK293T cells transfected with the vector expressing V5-FLAG-Nsp10, 14, 16, or empty vector along with ISRE-driven firefly luciferase and TK-driven renilla luciferase reporter vectors were un-treated or treated with IFN-α. **(C)** HEK293T cells transfected with the vector expressing V5-FLAG-Nsp10, 14, 16, or empty vector along with NF-κB-driven firefly luciferase and TK-driven renilla luciferase reporter vectors were un-treated or treated with TNF-α. Luciferase activity (firefly/renilla) in these cells was measured and normalized to the empty vector. Results were calculated from 3 technical repeats and presented as mean +/- standard error of the mean (SEM). (* $p < 0.05$; ** $p < 0.01$; *** $p < 0.001$; **** $p < 0.0001$; by one-way ANOVA and Tukey's multiple comparison test).

Fig S2

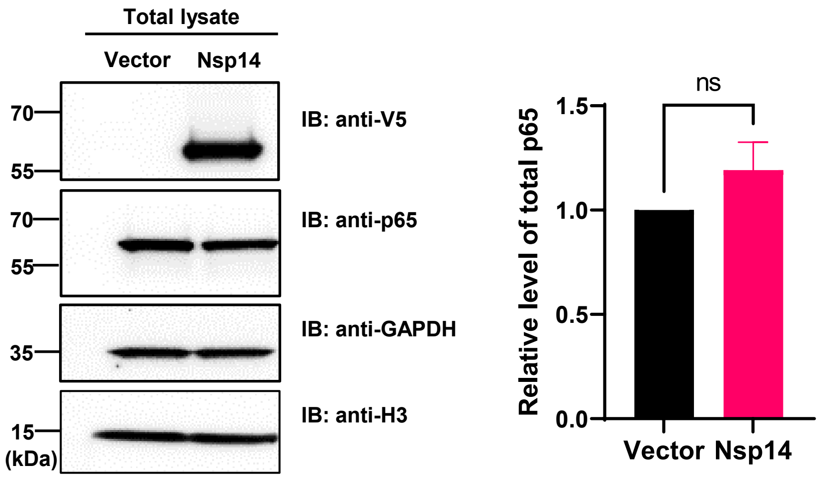

Total lysate
Vector
Nsp14
70
55
70
55
35
15
(kDa)
IB: anti-V5
IB: anti-p65
IB: anti-GAPDH
IB: anti-H3
ns
Relative level of total p65
1.5
1.0
0.5
0.0
Vector
Nsp14

Fig S2. NF-κB p65 in the total lysate of HEK293T cells transfected with the vector expressing V5-FLAG-Nsp14 or empty vector was analyzed by protein immunoblotting. Histone H3 was used as the nuclear marker. The intensity of the p65 protein band was quantified and normalized to the empty vector. (ns: not significant)

Fig S3

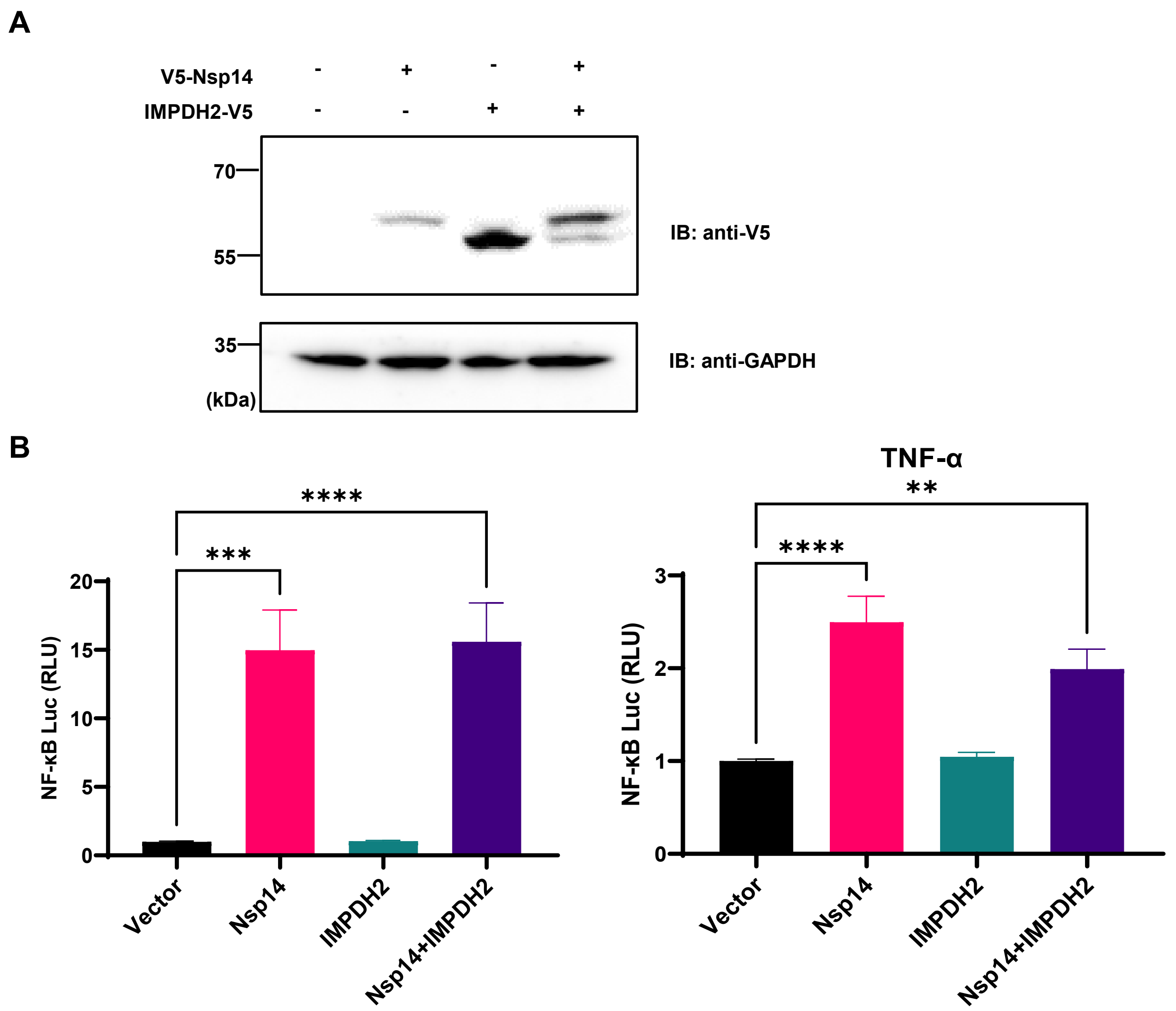
A
V5-Nsp14
IMPDH2-V5
70
55
35
(kDa)
IB: anti-V5
IB: anti-GAPDH
B
TNF-α
NF-κB Luc (RLU)
****
***
**
Vector
Nsp14
IMPDH2
Nsp14+IMPDH2

Fig S3. (**A**) HEK293T cells were transiently transfected with the vector expressing V5-Nsp14 or V5-IMPDH2, alone or together. Protein expression was analyzed by immunoblotting. (**B**) Cells in (**A**) were further transfected with NF-κB-driven firefly luciferase and TK-driven renilla luciferase reporter vectors without or with TNFα stimulation. Luciferase activity (firefly/renilla) was measured and normalized to the empty vector. Results were calculated from 3 independent experiments and presented as mean +/- standard error of the mean (SEM). (** $p < 0.01$; *** $p < 0.001$; **** $p < 0.0001$; by one-way ANOVA and Tukey's multiple comparison test)

Fig S4

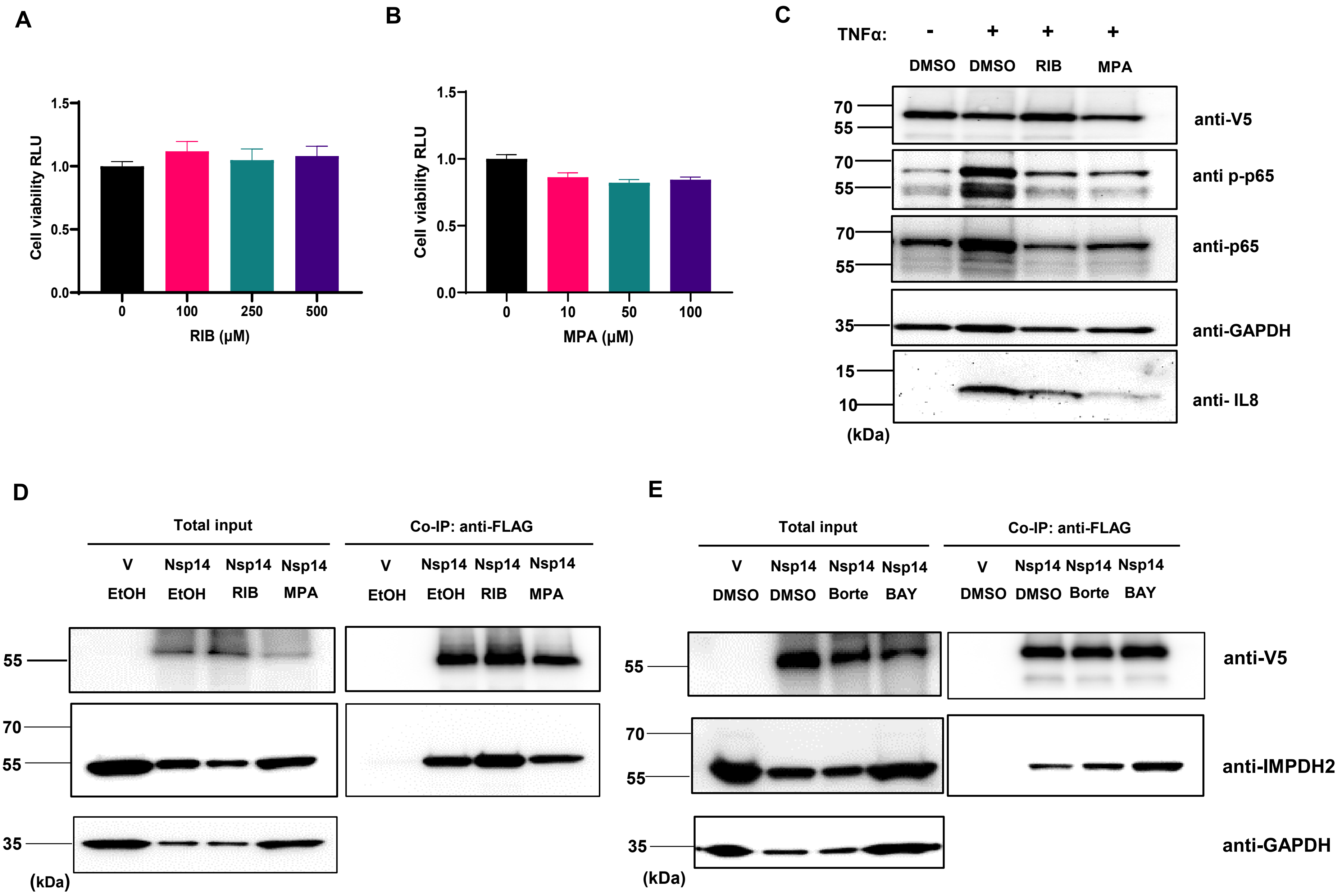
A
Cell viability RLU
1.5
1.0
0.5
0.0
0
100
250
500
RIB (μM)
B
Cell viability RLU
1.5
1.0
0.5
0.0
0
10
50
100
MPA (μM)
C
TNFα: - + + +
DMSO DMSO RIB MPA
70
55
anti-V5
anti p-p65
anti-p65
35
anti-GAPDH
15
10
anti- IL8
(kDa)
D
Total input
Co-IP: anti-FLAG
V Nsp14 Nsp14 Nsp14
EtOH EtOH RIB MPA
V Nsp14 Nsp14 Nsp14
EtOH EtOH RIB MPA
55
70
35
(kDa)
E
Total input
Co-IP: anti-FLAG
V Nsp14 Nsp14 Nsp14
DMSO DMSO Borte BAY
V Nsp14 Nsp14 Nsp14
DMSO DMSO Borte BAY
anti-V5
anti-IMPDH2
anti-GAPDH
(kDa)

Fig S4. (A, B) Viability of HEK293T cells treated with RIB (**A**) or MPA (**B**) for 24h at increasing doses was measured by CellTiter-Glo®. (**C**) A549 cells were transfected with the FLAG-V5-Nsp14 vector, followed with the treatment of IMDPH2 inhibitors (RIB 500 μM; MPA 100 μM) along with TNF-α stimulation for 24h. Cells were harvested and lysed for protein immunoblotting. **(D, E)** HEK293T cells were transiently transfected with the empty vector or FLAG-V5-Nsp14 vector, along with treatment of IMPDH2 inhibitors (**D**, RIB 500 μM, MPA 100 μM) or NF-κB inhibitors (**E,** bortezomib (Borte) 10 μM, BAY 11-7082 (BAY) 10 μM) for 24h. Cell lysates were prepared and subjected to protein co-IP assays using an anti-FLAG antibody. Precipitated protein samples were analyzed by immunoblotting using anti-V5 and anti-IMPDH2 antibodies.

Fig S5

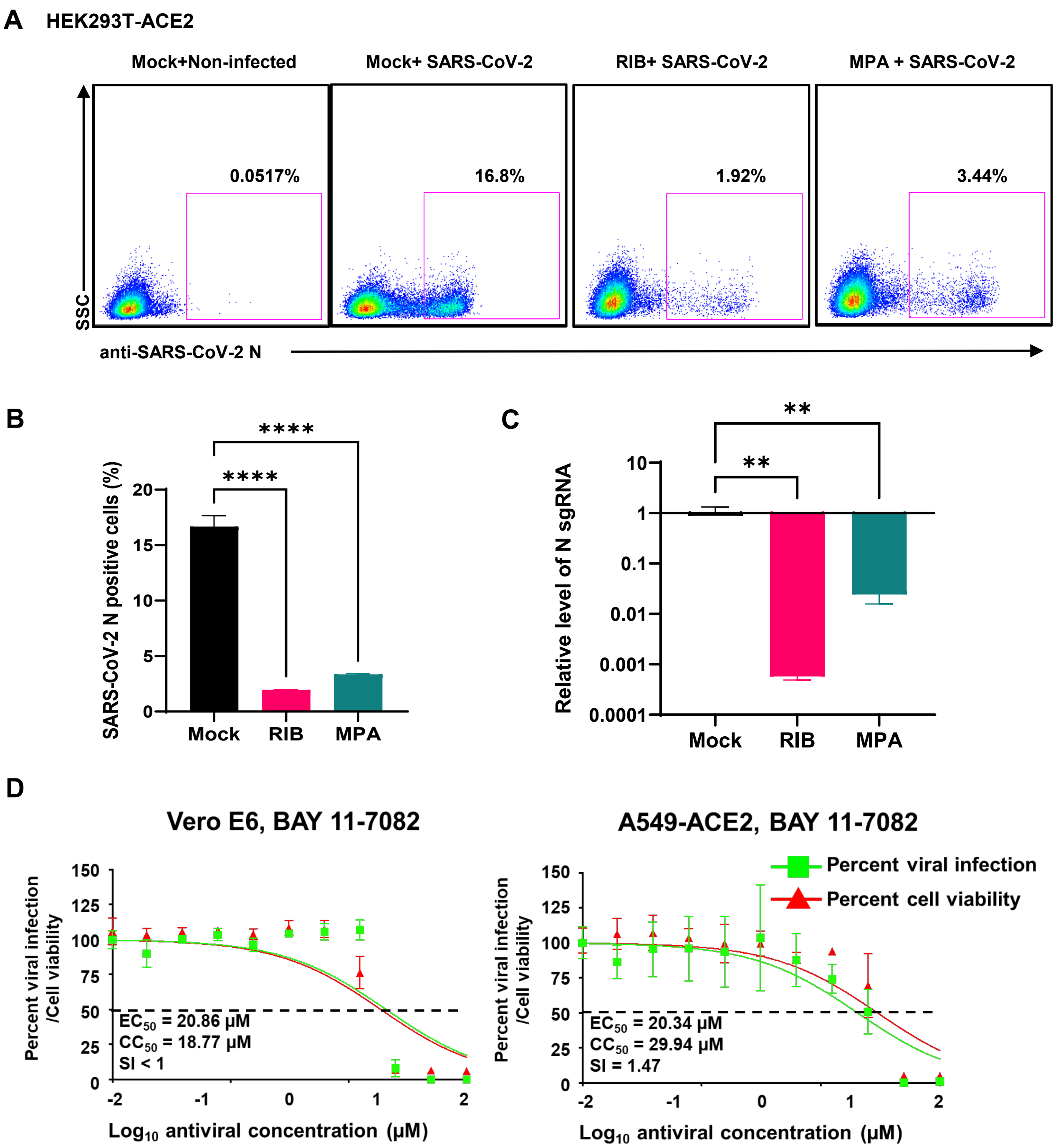

A HEK293T-ACE2
Mock+Non-infected
Mock+ SARS-CoV-2
RIB+ SARS-CoV-2
MPA + SARS-CoV-2
0.0517%
16.8%
1.92%
3.44%
SSC
anti-SARS-CoV-2 N
B
SARS-CoV-2 N positive cells (%)
****
****
Mock
RIB
MPA
C
Relative level of N sgRNA
**
**
Mock
RIB
MPA
D
Vero E6, BAY 11-7082
A549-ACE2, BAY 11-7082
Percent viral infection /Cell viability
Percent viral infection
Percent cell viability
EC50 = 20.86 μM
CC50 = 18.77 μM
SI < 1
EC50 = 20.34 μM
CC50 = 29.94 μM
SI = 1.47
Log10 antiviral concentration (μM)

Fig S5. HEK293T-ACE2 cells were treated with RIB (500 μM), or MPA (100 μM), or mock, and simultaneously infected with SARS-Cov-2 viruses for 24 h. The SARS-CoV-2 infection was detected by intracellular staining of SARS-CoV-2 N protein (**A**). The percentage of SARS-CoV-2 N protein-positive cells was calculated (**B**). Cells were harvested for RNA extraction, and N protein sgRNA was analyzed and normalized to the mock treatment (**C**). The results were calculated from 3 technical repeats and presented as mean +/- standard error of the mean (SEM). (** $p < 0.01$; **** $p < 0.001$; by one-way ANOVA and Tukey's multiple comparison test) (**D**) Vero E6 or A549-ACE2 cells were briefly infected with SARS-CoV-2 (~100 PFU/well), followed by treatment of BAY 11-7082. At 24 hpi, the cells were subjected to PRMNT assay at four biological replicates. Results were calculated as mean +/- standard deviation (SD). The dotted line indicates the 50% inhibition. Selectivity index (SI) is presented as CC_{50}/EC_{50}.
